# New Insights into Food Fermentation

**DOI:** 10.3390/foods11030283

**Published:** 2022-01-21

**Authors:** Juliano De Dea Lindner, Valentina Bernini

**Affiliations:** 1 Food Technology and Bioprocess Research Group, Department of Food Science and Technology, Federal University of Santa Catarina (UFSC), Florianópolis 88034-000, Brazil; 2 Department of Food and Drug, University of Parma (UNIPR), 43124 Parma, Italy

Food fermentation has been used for thousands of years for food preservation. At present, fermented foods remain appreciated by consumers thanks to the high-quality standards achieved, and the improvements in terms of nutritional and organoleptic characteristics. The production processes, type of raw material, microbial cultures, etc., can affect the quality and safety characteristics of these products. A vast array of microorganisms can be found in fermented foods, and microbial succession during fermentation, as well as during ripening, contributes to the desired properties of these foods. In addition to the sensory and safety aspects, microorganisms present in fermented foods can positively affect the health of people due to their potential probiotic nature and the production of beneficial metabolites such as vitamins and antioxidant compounds.

The goal of this Special Issue was to broaden the current knowledge on advanced approaches concerning food fermentation, gathering studies on conventional and unconventional food matrix fermentation, functional compounds obtained through fermentation, fermentations increasing quality and safety standards, as well as papers presenting innovative approaches shedding light on the microbial community that characterizes fermented foods. This Special Issue brings a series of 13 articles related to new insights into food fermentation.

Two review articles provide information concerning traditional fermented beverages. The first presents an updated view on Mexican beverages, systematizing information on the diversity, spatial distribution, and cultural history of beverages. Ojeda-Linares et al. [1] identified 16 Mexican fermented beverages (named *mescal*, *pulque*, *tejuino*, *pozol*, *chorote*, *colonche*, *saká*, *sendechó*, *balché*, *atole agrio*, *pox*, *sambudia*, *tesgüino*, *tepache*, *tuba*, and *taberna*) and 143 plant species involved in their production as substrates for fermentation. The authors highlight that microbial communities from backslopping have only scarcely been covered in studies of beverages such as *mescal*, *pulque*, and *atole agrio*. The second review addresses the properties, processing methods, microorganisms, and microbial dynamics issues regarding Ethiopian alcoholic beverages. In Ethiopia, the preparation and consumption of cereal- and fruit-based spontaneous, natural, and uncontrolled fermented alcoholic beverages are common. Cereal-based (*tella*, *borde*, *shamita*, *korefe*, *cheka*, and *keribo*) and fruit-based (*tej*, *ogol*, and *booka*) are described by Getachew Fentie et al. [2] as being the popular beverages in the country. Yeasts and lactic acid bacteria (LAB) are the predominant microorganisms encountered during fermentation. These beverages also contain significant amounts of total polyphenols and antioxidants. The alcohol contents and pH values of these beverages range from 1.53% to 21.7% and 2.9 to 4.9, respectively.

Many foods are still produced following traditional practices, although novel approaches to food fermentation have also attracted the interest of researchers and industries. Innovative technological and biological processes, as well as novel approaches of investigation, deeply interact to steer traditional products into modern diets and to open perspectives for the fermentation of unconventional substrates and food byproducts. The three papers submitted that dealt with functional foods addressed issues related to fruit and cereal fermentation. The research conducted in Italy by Maisto et al. [3] evaluated the addition of four different potential probiotic strains (*Lactiplantibacillus plantarum* subsp. *plantarum* ATCC14917, *Lactobacillus delbrueckii* subsp. *bulgaricus* ATCC 11842, *Lactobacillus acidophilus* ATCC 4356, and *Lacticaseibacillus rhamnosus* ATCC 7469) to date fruit-based fermented products (extruded snacks). After fermentation, changes in the polyphenol profile in terms of increased free phenolic compounds and related activity were observed. These results may be attributed to the metabolism of lactobacilli in catalyzing both the release of bioactive compounds from the matrix and the remodeling of polyphenolic composition in favor of more bioaccessible molecules. These positive effects were more evident when the snack was fermented with *L. rhamnosus*. The authors consider that the fermented snack may be proposed as a prototype of functional food, mainly indicated for athletics nutrition and supplementation.

The next two papers dealt specifically with the analysis of fermented cereals with heart health functional claims. Moon and Chang [4] fermented rice bran using a culture of *L. plantarum* EM, which exhibited significant cholesterol removal (45–68%) and strong antimicrobial activity against foodborne pathogenic bacteria and food spoilage fungi in vitro. Phytate levels were significantly reduced during fermentation by 53% due to the phytase activity of *L. plantarum*, indicating that fermented rice bran does not present nutrient-deficiency issues. The authors state that fermented rice bran is a promising low-cost functional food candidate and appears to satisfy consumer demands for environmental requirements concerning the re-utilization of biological byproducts. Yang et al. [5] explored the effects of different fermentation parameters on the quality of *natto* fermented with *Bacillus subtilis* GUTU09 and *Bifidobacterium animalis* subsp. *lactis* BZ25. Nattokinase activity, free amino nitrogen content, and sensory score were increased compared with control tests. The plate thrombolytic area and nattokinase activity increased significantly as the fermentation time increased, indicating that the *natto* exhibited strong thrombolytic action in vitro. Nattokinase is a fibrinolytic enzyme released by the *B. subtilis natto* bacteria during the fermentation process.

Despite recent innovations on “omic” techniques, including genomic approaches using high-throughput sequencing in combination with advanced metabolomics, there are many fermented products with limited information about their microbial diversity and dynamics (succession). This is particularly true for traditional products, which represent a rich niche for discoveries involving microorganisms more robust for industrial production. For example, the unknown microbiota can reveal new molecular mechanisms of quorum sensing and new bioactive molecules with beneficial effects.

The next four papers deal specifically with the analyses of the microbiome of fermented foods. The composition of the microbiota has an important impact on the quality and safety of products due to the growth and interaction between microorganisms during processing and ripening. Therefore, much effort has been made by the authors to applicate molecular tools and omic systems to investigate the microbial community composition of fermented foods. The paper presented by Jiang et al. [6] evaluated the effects of raw materials and fermentation periods on the microbial ecology of Chinese *paocai* using metagenomic analyses. *Paocai* is typically made by fermenting red radish or cabbage with brine for 6–10 days. *Secundilactobacillus paracollinoides* and *Furfurilactobacillus siliginis* were the characteristic bacteria in red radish *paocai*, whereas 15 species of characteristic microbes were found in cabbage *paocai*. Furthermore, the study also enabled the identification of volatile organic compounds (VOCs) and the establishment of their relationship to the microorganisms, which provided insights into the microbial flavor profiles of these fermented vegetables which are popular and traditional in Asian countries.

The microbiota of Protected Designation of Origin (PDO) cheeses play essential roles in defining their quality and typicity, and could be applied to protect these products from counterfeiting. Zago et al. [7] applied DNA meta-analysis to study the possible role of microbiota in distinguishing Italian *Grana Padano* (GP) cheese from generically hard cheeses (HCs). The microbial structure and the genotypic fingerprinting of the bacterial taxa of 119 GP samples were evaluated by 16S rRNA gene sequencing (DNA metabarcoding) and RAPD-PCR from total cheese DNA (RAPD-PCR metafingerprinting), respectively, and compared with 49 samples of generically HC from retail. The obtained metagenotypes were evaluated as possible tools to differentiate the two sampling groups, assuming, unlike metataxonomic analysis, that this technique was able to identify strain- or group-specific differences within complex microbial communities. Metataxonomic and metafingerprinting data were then used as inputs to train and validate a two-class (GP vs. HC) classifier based on a neural network as a computational model. Although metataxonomic data did not allow for reliable classification, the discriminatory power of metafingerprinting enabled the building of a robust model (very high binary accuracy). When trained with metagenotyping data, the model correctly classified the samples. The molecular (meta)fingerprint of the entire microbial community could be promising to assist GP authentication and to distinguish it from imitation products.

Degenhardt et al. [8] studied the presence of hepatitis E virus (HEV) and rotavirus-A (RV-A), as well as fungal and bacterial communities, using metagenomics and culture-dependent methods in artisanal *colonial* salami-type dry-fermented sausages from Brazil. LAB and yeasts dominated the microbiome. *Lactilactobacillus sakei* and *Debaryomyces hansenii* were ubiquitous and the most abundant species. The characteristics of the raw material and hygiene of the manufacturing process resulted in high loads of beneficial microorganisms and the absence of HEV and RV-A viruses, as determined by RT-qPCR assays. The wide array of traditional fermented sausages worldwide represents a reservoir of microbial biodiversity and can be an important source of new biotechnological strains able to preserve typical features lost due to the introduction of commercial cultures. Particularly, in recent decades, the use of starter cultures has been introduced in the meat industry to guide fermentation, enhancing product safety but losing biodiversity and peculiar characteristics to the products in terms of both technological and organoleptic traits. Barbieri et al. [9] analyzed 15 artisanal salamis from the Mediterranean area (Italy, Spain, Croatia, and Slovenia) to evaluate the microbiota composition through culture-dependent and culture-independent techniques. LAB and coagulase-negative cocci were the dominant populations, with the highest LAB presence in Croatian and Italian samples. Metagenomic analysis showed high variability in microbial composition: *L. sakei* was the dominant species, but *Companilactobacillus* spp. was present in high amounts (45–55% of the total amplicon sequence variant) in some Spanish sausages. *Staphylococcus epidermidis*, *Staphylococcus equorum*, *Staphylococcus saprophyticus*, *Staphylococcus succinus*, and *Staphylococcus xylosus* were detected. The growth and survival of different microbial groups in the sausages reflect in the first-instance safety characteristics (i.e., biogenic amine concentration). In addition, the volatilome, and consequently, the peculiar sensory traits of traditional products, are dependent on the complexity of the microbiota. In many sausages considered in this paper, increased microbial biodiversity caused VOCs to be more complex, both qualitatively and quantitatively.

Due to the increasing occurrences of worldwide food-borne disease outbreaks caused by biogenic amines, food safety has received more concern in the production of fermented meat products. The composition of the microbiota has a direct impact on the safety of products. Using high-throughput sequencing, culture-dependent, and HPLC methods, Ma et al. [10] investigated the contribution and regulation of biogenic amines (BAs) by dominant microorganisms during traditional fish sauce fermentation in China. *Tetragenococcus* (40.65%), *Lentibacillus* (9.23%), *Vagococcus* (2.20%), *Psychrobacter* (1.80%), *Pseudomonas* (0.98%), *Halomonas* (0.94%), and *Staphylococcus* (0.16%) were the dominant genera observed. The contents of BAs gradually increased as the fermentation progressed. After 12 months of fermentation, the histamine content (55.59 mg/kg) exceeded the toxic dose recommended by the United States Food and Drug Administration (FDA). Correlation analysis showed that the microbiota made a considerable contribution to the accumulation of BAs. *Staphylococcus nepalensis* 5-5 and *S. xylosus* JCM 2418 strains with a high BA reduction ability were screened out of 44 low BA-producing dominant isolated strains and might be potential functional cultures for BA control in meat. Yu et al. [11] explored the influences of thyme (*Thymus vulgaris* L.) on the growth, gene expression, and histamine accumulation by *Proteus bacillus* isolated from Xinjiang smoked spontaneous fermentation horsemeat sausage, a popular appetizer in China. RT-qPCR was employed to evaluate the gene expression level of histidine decarboxylase (HDC) cascade-associated genes. Histamine accumulation was suppressed by inhibitory effects of the thyme microcapsule on histamine-producing bacteria and reductions in the transcription of hdcA and hdcP genes. Furthermore, the addition of thyme microcapsules in Xinjiang smoked horsemeat sausage inhibits the potential spoilage and pathogenic microbial growth (e.g., Enterobacteria).

Jiang et al. [12] investigated the effect of lipase addition on *suanzhayu*, a Chinese traditional solid fermented fish product, which is produced by mixing rice powders with seasonings and fresh fish meat in a sealed long fermentation condition. The addition of lipase had little effect on the structure of the microbiome, but promoted the growth of *Proteus* and the formation of VOCs, especially aldehydes and esters. The correlation analysis showed that *Lactobacillus*, *Enterococcus*, and *Proteus* played an important role in the safety of the product, inhibiting the potential pathogenic *Escherichia*-*Shigella*. The addition of lipase could be used as a novel means to enhance the quality of *suanzhayu*. Finally, the last paper evaluated lactic acid fermentation of *Arthrospira platensis* biomass, focusing on changes in the aromatic profile of this cyanobacterium widely used in food formulations and mainly consumed as a food supplement because of its high nutritional value. Martelli et al. [13] applied two different stabilization treatments on the biomass (UV light treatment and sterilization) prior to solid-state fermentation with *Lacticaseibacillus casei* 2240 and *L. rhamnosus* GG. The fermentation process was useful for off-flavor reduction. In particular, the fermentation process significantly influenced the concentration of those compounds responsible for aldehydic/ethereal, buttery/waxy (acetoin and diacetyl), alkane, and fermented aromatic notes (isoamyl alcohol). Fermentation with LAB can be an interesting tool to obtain a lyophilized spirulina powder with more pleasant sensory properties for potential use as a food supplement or in food formulations.
